# Identification of stable normalization genes for quantitative real-time PCR in porcine articular cartilage

**DOI:** 10.1186/2049-1891-3-36

**Published:** 2012-11-12

**Authors:** Ryan S McCulloch, Melissa S Ashwell, Audrey T O’Nan, Peter L Mente

**Affiliations:** 1 Joint Department of Biomedical Engineering, North Carolina State University, Raleigh, NC, USA; 2 University of North Carolina, Chapel Hill, North Carolina, USA; 3 Animal Science Department, North Carolina State University, Raleigh, NC, USA

**Keywords:** Cartilage, Housekeeping, Normalization, Porcine, Reference, Stability

## Abstract

**Background:**

Expression levels for genes of interest must be normalized with an appropriate reference, or housekeeping gene, to make accurate comparisons of quantitative real-time PCR results. The purpose of this study was to identify the most stable housekeeping genes in porcine articular cartilage subjected to a mechanical injury from a panel of 10 candidate genes.

**Results:**

Ten candidate housekeeping genes were evaluated in three different treatment groups of mechanically impacted porcine articular cartilage. The genes evaluated were: *beta actin*, *beta*-*2*-*microglobulin*, *glyceraldehyde*-*3*-*phosphate dehydrogenase*, *hydroxymethylbilane synthase*, *hypoxanthine phosphoribosyl transferase*, *peptidylprolyl isomerase A* (*cyclophilin A*), *ribosomal protein L4*, *succinate dehydrogenase flavoprotein subunit A*, *TATA box binding protein*, and *tyrosine 3*-*monooxygenase*/*tryptophan 5*-*monooxygenase activation protein*—*zeta polypeptide*. The stability of the genes was measured using geNorm, BestKeeper, and NormFinder software. The four most stable genes measured via geNorm were (most to least stable) *succinate dehydrogenase flavoprotein*, *subunit A*, *peptidylprolyl isomerase A*, *glyceraldehyde*-*3*-*phosphate dehydrogenase*, *beta actin*; the four most stable genes measured via BestKeeper were *glyceraldehyde*-*3*-*phosphate dehydrogenase*, *peptidylprolyl isomerase A*, *beta actin*, *succinate dehydrogenase flavoprotein*, *subunit A*; and the four most stable genes measured via NormFinder were *peptidylprolyl isomerase A*, *succinate dehydrogenase flavoprotein*, *subunit A*, *glyceraldehyde*-*3*-*phosphate dehydrogenase*, *beta actin*.

**Conclusions:**

BestKeeper, geNorm, and NormFinder all generated similar results for the most stable genes in porcine articular cartilage. The use of these appropriate reference genes will facilitate accurate gene expression studies of porcine articular cartilage and suggest appropriate housekeeping genes for articular cartilage studies in other species.

## Background

With relative quantitative real-time reverse transcriptase PCR (qPCR), multiple genes across many specimens may be evaluated to measure changes in expression. However, to accurately determine the relative expression levels, and the corresponding fold changes, a reference gene is necessary. Reference genes, frequently termed “housekeeping genes,” are used to normalize the expression results for differences in cDNA quantity between different specimens and thus enable comparisons between genes of interest across treatments. In order to act as a reference, a housekeeping gene’s expression should remain unchanged regardless of treatment. Genes whose expression is generally unchanged with treatment conditions are most often associated with basic cellular processes such as metabolism. Our goal was to identify the most appropriate reference genes for analyses of porcine articular cartilage.

Regardless of the tissue being examined, housekeeping genes have usually been selected based on genes used in previous studies in various human tissues, and typically include *beta actin* (*actb*), *beta2microglobulin* (*b2m*), *glyceraldehyde3phophate dehydrogenase* (*gapdh*), *hydroxymethylbilane synthase* (*hmbs*), *hypoxanthine guanine phosphoribosyl transferase* (*hprt*), *ribosomal protein L13a* (*rpl13a*), *ribosomal protein S18* (*s18*), *succinate dehydrogenase flavoprotein subunit A* (*sdha*), *TATA box binding protein* (*tbp*), and *tyrosine 3monooxygenase*/*tryptophan 5monooxygenase activation protein*—*zeta polypeptide* (*ywhaz*). A variety of genes have been used in the past as housekeeping genes in cartilage studies in various species. *Gapdh* has been used as a housekeeping gene in studies of human, bovine, porcine, and caprine articular cartilage, including both normal and osteoarthritic (OA) samples [1,2]. Swingler *et al*. [3] used *sdha* as a reference gene in their study of human OA cartilage. These genes appeared to be selected based on literature, not selected based on evaluation of a panel of genes to identify the most stable gene. Pombo-Suarez *et al*. [4] evaluated nine of these same reference genes in addition to *ubiquitin C* in human cartilage with advanced OA and found the rarely used housekeeping genes *TATA box binding protein* (*tbp*), *ribosomal protein L13a* (*rpl13a*) and *beta2microglobulin* (*b2m*) to be the most stably expressed genes while they found the most commonly used genes (*gapdh*, *actb* and *18s*) to be the least stable. Pombo-Suarez *et al*. [5] therefore recommended that *tbp*, *rpl13a* and *b2m* be used as housekeeping genes for human cartilage research. In a canine study of normal and OA cartilage, *rpl13a* and *sdha* were identified as the most stable reference genes [6]. The pig has been used as a model of human OA disease, cartilage repair, xenotransplantation, and gene transfer research, but no one has yet conducted a study to determine the ideal reference gene(s) for gene expression studies in porcine articular cartilage.

The reason for using a reference gene is to control for differences in the amount of starting material, efficiency of amplification, and differences in expression from cells and the overall level of transcription [7]. Therefore, selecting a stable housekeeping gene presents a circular problem: determining a stable gene when that gene is expressed differently across samples/tissues. Several methods have been developed to identify the best housekeeping gene(s) from an initial panel of potential reference genes. Three of the most commonly used methods are geNorm, BestKeeper, and NormFinder. All of these programs attempt to provide a relative measure of the stability of a panel of genes by comparing their individual stability in relation to that of the entire panel.

In geNorm [8], the average pairwise gene expression variation of each potential housekeeping gene is compared to all other evaluated reference genes. The genes that demonstrate the least variance in comparison with all other genes are ranked as the most stable genes and are therefore likely to be the best reference genes. The authors developed a Visual Basic Application for Microsoft Excel (geNorm; [8]) to carry out the analysis.

BestKeeper, developed by Pfaffl *et al*. [9], uses an Excel based application to determine the most stable gene from a panel of up to ten candidate genes. The geometric mean of the cycle threshold values (Ct values) for each sample across all housekeeping genes are combined together to form the BestKeeper index. Subsequently, each individual gene is compared in a pair-wise fashion via Pearson correlation coefficients to the BestKeeper index. The outcome is a ranked order of genes in terms of their stability. The highest ranked gene is the most stable. Rather than using only one housekeeping gene or the impractical method of using all potential housekeeping genes, the authors recommended the use of the best 3 or 4 genes as that provides a realistic number of housekeeping genes while still providing adequate normalization of results.

NormFinder was developed by Ohl *et al*. [10] and also uses an Excel based application to determine the most stable genes from a panel. This program uses a model-based approach, where all expression values are compared via analysis of variance, and all genes and specimen results are utilized for estimation of the expected expression values. A stability measure is calculated to identify the genes that deviate the least from the calculated values [11].

Nygard *et al*. [12] evaluated a panel of nine genes using the geNorm approach to determine the best housekeeping genes across 17 different porcine tissues. That study included tissues such as muscle, adipose, heart, bladder, kidney, liver, skin, intestine, pancreas, bone marrow, and different portions of the brain, but no cartilage. They identified *actb*, *ribosomal protein L4* (*rpl4*), *tpb*, and *hprt* as the most stably expressed housekeeping genes across the 17 tested tissues. Though cartilage was not included in the set of tissue they evaluated, their set of potential housekeepers included all of the genes previously discussed as commonly used in cartilage in other species with the exception of *rpl13a* and *18s*. *Rpl13a*, like *rpl4*, encodes a protein of the 60S subunit of ribosomes and is still a good candidate while *18s* has been shown to vary in proportion to total RNA and is therefore no longer considered a good candidate for normalization [9]. In this study we propose to determine the best housekeeping genes for use in porcine articular cartilage and to evaluate three software packages, geNorm, BestKeeper, and NormFinder for determining overall gene stability. We used the nine genes identified by Nygard and co-workers [12] as potential housekeeping genes as a starting point with the addition of *peptidylprolyl isomerase A* (*ppia*). *Ppia* was added because it has been used as a normalizing gene in cartilage for both OA-related [13,14] and non-OA related studies [15,16] and it exhibited no differential expression in impacted and control cartilage specimens in our previous work [17].

## Methods

RNA was extracted from the articular cartilage of 40 porcine patellae obtained from an *in vitro* study examining gene expression changes following an applied impact injury. Patellae were subjected to one of three treatments—axial impaction, shear impaction or no impaction (non-impacted control)—and were maintained in culture for 0 (no culture), 3, 7 or 14 d. The expression of ten potential housekeeping genes: *actb*, *b2m*, *gapdh*, *hmbs*, *hprt*, *ppia*, *rpl4*, *sdha*, *tbp*, and *ywhaz* were evaluated using quantitative real-time PCR (qPCR). The relative stability of the genes was evaluated using BestKeeper [9], NormFinder [10] and geNorm [8].

### Tissue collection

Porcine knee joints were obtained from a local slaughterhouse. Patellae were sterilely removed from the joint and assigned to one of three treatment groups: control, axial impacted, or shear impacted. Patellae to be impacted were positioned in a custom holder in a hydraulic load frame (MTS MiniBionix, MTS, Minneapolis, MN). Impactions were carried out with a stainless steel impactor measuring 10mm long by 10 mm in diameter. For the axial impactions, a 2,000 Newton load was rapidly applied (loading rate of 25 mm/s) normal to the patella surface in the center of each facet. For the shear impaction a 500 Newton axial load was slowly applied (loading rate of 0.5 mm/s) followed by a rapid (200 mm/s) 10mm horizontal displacement to induce larger shear forces. Intact patellae were then placed in culture at 37°C with 5% CO_2_. Media (Delbecco’s/MEM, 10% fetal bovine serum, ascorbic 2-Phosphate (25 μg/mL), penn 100 units/ml –strep 100 μg/ml- amphotericin B 25 μg/mL; Gibco, Grand Island, NY) was changed daily. Patellae were maintained in culture for 0, 3, 7, or 14 d at which point 5 mm × 10 mm full thickness sections of cartilage were removed and immediately flash frozen in liquid N_2_ and stored at -80°C until RNA extraction was performed. Zero day tissue was harvested on the day of impaction approximately 2 hours after impaction.

### RNA extraction

Total RNA was extracted by first grinding the cartilage specimens in a mortar and pestle cooled by liquid nitrogen. The resulting powder was dissolved in Tri Reagent (Molecular Research Center Inc., Cincinnati, OH). The tissue was then homogenized in a BeadBeater® (Biospec Products, Bartlesville, OK) for 10 s at 4,800 oscillations per minute. The manufacturer’s protocol was followed except the RNA was first precipitated in the presence of acetic acid and then in the presence of ammonium acetate [18-20]. Finally, on-column DNAse digestion was accomplished with an RNeasy kit (Qiagen, Valencia, CA). Purity of the RNA was measured on a Nanodrop spectrophotometer (Thermo Scientific, Wilmington, DE) and a sampling of samples were run on a 1% agarose gel to ensure little to no degraded RNA.

### qPCR

A High Capacity cDNA reverse transcription kit (Applied Biosystems Inc., Foster City, CA) was used to reverse transcribe 250 ng of total RNA per the manufacturer’s protocol. Subsequently, reactions were diluted 1:10 to provide enough template for all genes to be evaluated. PCR primer sequences for the evaluated genes were obtained from Nygard *et al*. [12] with the exception of *ppia* (NM_214353.1). The *ppia* primers were designed with Beacon Designer software (Premier Biosoft Intl., Palo Alto, CA) from porcine gene sequences as previously described (F: 5’-GCAGACAAAGTTCCAAAGACAG-3’, R: 5’-AGATGCCAGGACCCGTATG-3’) [17] spanning an intron to detect genomic contamination.

qPCR was performed in a volume of 20 μL, consisting of 1 μL of diluted cDNA, 400 nmol/L of forward and reverse primers, 10 nmol/L fluorescein, and 1X Power SYBR Green Master Mix. A three-step amplification protocol was performed in an iCycler IQ (Bio-Rad, Hercules, CA); an initial denaturation was performed with one cycle at 95°C for 7 min. Subsequently, target amplification involved 40 cycles of 30 s at 95°C, 30 s at 56°C to 62°C for annealing, then extension for 30 s at 72°C. After 40 amplification cycles, PCR products were evaluated for quality using melt curve analysis, which entailed 5 min at 72°C, 1 min at 95°C, and 1 min at 55°C. Reactions were performed in duplicate and Ct values were averaged for the replicates and negative controls were included to detect contamination.

Standard curves were evaluated for each primer by combining equal amounts of cDNA from each specimen into a pool. The pool was then diluted in serial dilutions of 1:3, 1:9, 1:27, 1:81, and 1:243. The dilutions were evaluated in triplicate by iCycler iQ Real-Time PCR Detection System Software v3.1 (Bio-Rad, Hercules CA) to calculate amplification efficiency.

### Data analysis

BestKeeper, geNorm, and NormFinder were used to select the most stable genes. For the BestKeeper program, raw Ct values were entered and a BestKeeper index, which is the geometric mean of all housekeeping gene Ct values, was calculated. Pearson correlations between each individual gene and the BestKeeper index were calculated and reported as the BestKeeper correlation coefficient. Genes with the highest BestKeeper correlation coefficient were considered the most stably expressed. While there is no specific threshold for the BestKeeper correlation coefficient, Pfaffl *et al*. [9] recommended the use of multiple genes geometrically averaged to control for outliers. They suggested three genes were a realistic number to use for most studies while still ensuring accurate normalization [9].

The geNorm program uses normalized Ct values, where Ct values for a particular gene are normalized to the specimen with the highest expression (minimum Ct value) for that gene. The normalized Ct values (Q) are calculated via the delta-Ct formula (Equation 1).

(1) $$Q=E^{\left(min\;Ct-sampleCt\right)}$$

where:

Q = normalized Ct value for a given gene in the current specimen,

E = PCR amplification efficiency (ranging from 1 to 2 with 100% = 2) calculated from standard curve,

minCt = minimum Ct value for the gene among all specimens, and

sampleCt = the Ct value of the gene for the current specimen.

In geNorm, pairwise comparisons of each gene with every other gene are performed to determine their relative stability in gene expression. Vandesompele *et al*. defined the stability measure M_j_ of a given gene (j), as the mean of all pairwise variations V_jk_, between gene j and all other examined genes [7] (Equation 2).

(2) $$M_{j}=\frac{\sum_{k=1}^{n}V_{\mathrm{jk}}}{n-1}$$

where:

M_j_ = gene stability measure,

V_jk_ = pairwise variation of gene j relative to gene k, and

n = total number of number of examined genes.

Lower M values represent genes with more stable expression across specimens being compared.

NormFinder also relies on Q values (Equation 1) as input, calculated from the Ct values. The program then log transforms the data and a model based approach is used with analysis of variance to calculate the expected value for each sample. The deviation of the measured value from the expected is used to calculate a stability value that ranks the genes, with the lowest value indicating the most stable [10,11].

Vandesompele *et al*. [7] suggested using 3 or 4 of the most stable genes for accurate normalization, using the geometric mean of the Ct values of the chosen housekeeping genes (Equation 3).

(3) $$Geometric\;mean=\sqrt[{n}]{a_{1}a_{2}\ldots a_{n}}$$

where:

a = individual Ct values for the specimen’s housekeeping genes and

n = total number of housekeeping genes employed.

The geometric mean is better able to control for outliers and abundance differences than the arithmetic mean (the sum of the individual Ct values divided by the n - the total number of values). Thus the most accurate normalization strategy is to use the geometric mean of the 3 or 4 most stable genes for normalization [7,9].

## Results

Ten potential housekeeping genes for articular cartilage were evaluated in an *in vitro* porcine patella organ culture model that included non-impacted control, axially impacted and shear impacted tissue subjected to culture times of 0 (no culture), 3, 7, or 14 d. Two or more specimens at each impaction treatment/time point combination were evaluated using a total of 40 patellae (Table 1).

**Table 1:** Number of samples examined in each treatment group

**Culture time (days)**	**Impaction treatment**		
	**Axial**	**Shear**	**Control**
0	4	3	3
3	4	4	3
7	3	3	2
14	4	4	3
Total Samples:		40	

PCR amplification products were obtained for all genes but *hprt*, which was excluded from analysis as it displayed consistently high Ct values (greater than 35) and failed to amplify in five samples in which all other genes amplified, suggesting it is not expressed in sufficient quantity to be used as an effective housekeeping gene in these specimens. The Ct values from each of the candidate genes were input directly in the BestKeeper software [9] and were used to calculate the input values (Q values) for geNorm [8], and NormFinder [10].

**Figure 1:**
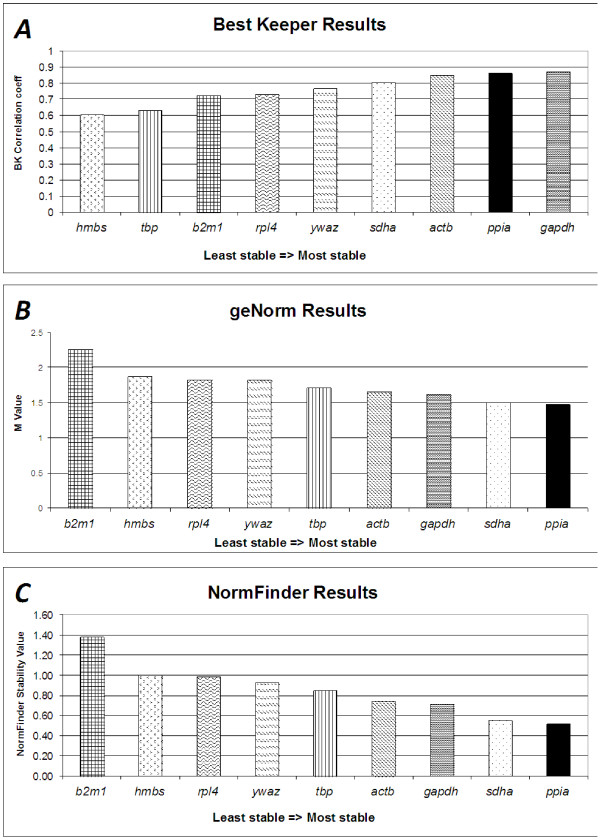
**Ranking results of most stable normalization genes.** (**A**) The BestKeeper results for candidate genes. BestKeeper correlation coefficient is ploted on the y-axis. A higher correlation coefficient corresponds to a more stably expressed gene. (**B**) The geNorm results for candidate genes. The M-value (y-axis) calculated by geNorm is a measure of stability of the gene expression across specimens. Genes with lower M-values show increased stability. (**C**) The NormFinder results for candidate genes. NormFinder stability values are plotted on the y-axis, with lower stability values indicating a gene that is more stable.

BestKeeper calculated the stability ranking of the nine genes to be (in order of most stable to least stable): *gapdh*, *ppia*, *actb*, *sdha*, *ywhaz*, *rpl4*, *b2m*, *tbp*, and *hmbs* (Figure 1A). The geNorm results differed slightly with a stability order of: *sdha*/*ppia* (*tied*), *actb*, *gapdh*, *tbp*, *ywhaz*, *hmbs*, *rpl4*, and *b2m* (Figure 1B). NormFinder ranked the stability as: *ppia*, *sdha*, *gapdh*, *actb*, *tbp*, *ywaz*, *rpl4*, *hmbs*, and *b2m1* (Figure 1C).

While there were differences in the ranked order, all three analysis programs found that the same four genes exhibited the highest stability in porcine cartilage across our three treatment groups and four time points. The most stably expressed genes were *gapdh*, *ppia*, *actb*, and *sdha*. Because 3 or 4 housekeeping genes are generally recommended [7-9], we suggest the geometric mean of *gapdh*, *ppia*, *actb*, and *sdha* is an appropriate choice for an accurate normalization strategy in porcine cartilage samples.

## Discussion

To make accurate comparisons of changes in gene expression when studying a tissue, it is important to select the best reference gene(s) for normalizing Ct values. A perfect reference gene would be expressed stably in all cells under all conditions, however, a perfect reference gene has not been found. BestKeeper, geNorm, and NormFinder provide three approaches for examining potential genes to select the most stable housekeeping genes for a given set of conditions.

We found all programs easy to use, each providing an easily accessible measure of gene expression stability in a tissue. The three programs agreed on the 4 most stable genes. The geNorm program provided a simpler more user-friendly and structured interface as it was programmed in Microsoft Visual Basic Language (VBL). This made geNorm a simpler program to use, however the equations were hidden from the user and missing values for a specimen for a particular gene could not be accepted, necessitating the removal of the entire specimen from the analysis. In addition, the user is required to calculate a Q value in geNorm, which may be an additional calculation for the user, depending upon the software associated with the qPCR instrument used. BestKeeper is based upon an Excel spreadsheet but does not employ VBL and therefore did not have as simple a user interface. However it allowed the user to clearly see the equations used and the various steps involved in the calculation. Additionally, BestKeeper allowed for direct input of Ct values, and accepted missing Ct values for a particular gene. NormFinder was an add-in for Excel and relied upon Q values as input like geNorm. Similarly to geNorm, NormFinder could not accept missing data in the input, and the equations used in the calculation of the stability value are hidden from the user. While all programs were relatively easy to use, BestKeeper was our preferred method because of its ability to handle missing data, the ease of entering Ct values directly, and the transparency of calculations in each step.

The candidate housekeeping genes that were evaluated in this study were selected from various studies examining gene expression in cartilage. The majority of these genes were also examined by Nygard *et al*. [12] in a study evaluating reference genes in 17 porcine tissues which showed that the ideal reference genes are tissue specific. Therefore, it is important to evaluate potential housekeeping genes for the particular tissue being utilized in a study. Because our research involves cartilage, the intent of this study was to build on the work of Nygard and colleagues to determine the most appropriate housekeeping genes specifically for porcine articular cartilage. *Ppia* was added because it has been used as a normalizing gene for other studies examining cartilage [13-15] and it exhibited no differential expression in impacted and control specimens in our previous work [17]. We found *ppia*, *sdha*, *gapdh*, and *actb* to be the most stable reference genes for porcine articular cartilage across our treatments and timepoints.

In addition to the Nygard *et al*. [12] study, four previous studies have evaluated reference genes for various porcine tissue. Erkens *et al*. [21] evaluated ten potential reference genes and found that *actb*, *tbp* and *topoisomerase 2beta* were stable and that *sdha* appeared to be unstable in porcine backfat and muscle. Four genes were analyzed by Svobodova *et al*. [22] in seven porcine tissues, including heart, liver, lungs, spleen, kidney and muscle. In contrast to the results of our study, *gapdh* was found to be relatively unstable while *hprt* was found to be stable. Kuijk *et al*. [23] studied seven reference genes in different stages of porcine embryonic development. Of the panel of genes, *gapdh*, *pgk1*, *s18* and *ubc* showed high stability. Nygard *et al*. [12], Svobodova *et al*. [22], Piorkowska *et al*. [24], and Erkens *et al*. [21] found tissue specific regulation of potential reference genes. Therefore our study was critical for identifying the best reference genes specifically for articular cartilage.

Previous gene expression studies in pig, cattle, goat, sheep, dog and human cartilage [1,2,25,26] have used a variety of housekeeping genes, including *gapdh*, *sdha*, *s18* and *actb*, but these genes appeared to be selected based on what others had used in similar studies and not because they had been selected as the most stably expressed gene, as we have done here. Evaluation of appropriate housekeeping genes in human cartilage with advanced OA has been reported by Pombo-Suarez and co-workers [5] who suggest that *tbp*, *rpl13a*, and *b2m* be used in such studies. *Tbp*, *rpl4* (which is similar in function to *rpl13a*), and *b2m* did not perform as well in our panel of genes with *b2m* generally being the least stable of the genes we examined. While we found *ppia*, *sdha*, *gapdh*, and *actb* to be the most stable, Pombo-Suarez *et al*. [4] found that *gapdh* in particular was one of the least stable genes while *actb* and *sdha* were in the middle of the pack. Differences in the selection of housekeeping genes for cartilage between Pombo-Suarez *et al*. and this study could represent expression differences due to species (human vs. porcine), tissue condition (*in vivo* normal and OA tissue vs. *in vitro* impacted and control), or even age (sample averages from human tissue ranged from 72 to 81 yr).

Using the pig as a model for cartilage repair studies and osteoarthritis research is quite common today. Therefore we think our results will be useful to researchers evaluating gene expression in porcine articular cartilage and as a starting point for choosing appropriate housekeeping genes in other species.

## Conclusions

BestKeeper, geNorm, and NormFinder all generated similar results for the most stable genes in porcine articular cartilage. *Peptidylprolyl isomerase A*, *succinate dehydrogenase flavoprotein*, *subunit A*, *glyceraldehyde*-*3*-*phosphate dehydrogenase* and *beta actin* should be used together by taking the geometric mean of the expression to effectively normalize expression levels for the gene of interest. The use of these appropriate reference genes will facilitate accurate gene expression studies of porcine articular cartilage and will facilitate the choice of appropriate housekeeping genes for articular cartilage studies in other species.

## Abbreviations

(actb): Beta actin; (b2m): Beta-2-microglobulin; (cycle threshold): Ct; (gapdh): Glyceraldehyde-3-phophate dehydrogenase; (hmbs): Hydroxymethylbilane synthase; (hprt): Hypoxanthine guanine phosphoribosyl transferase; (osteoarthritis): OA; (ppia): Peptidylprolyl isomerase A; (quantitative real-time polymerase chain reaction): qPCR; (rpl13a): Ribosomal protein L13a; (s18): Ribosomal protein S18; (sdha): Succinate dehydrogenase flavoprotein subunit A; (tbp): TATA box binding protein; (ywhaz): Tyrosine 3-monooxygenase/tryptophan 5-monooxygenase activation protein—zeta polypeptide.

## Competing interests

The authors declare that they have no competing interests.

## Authors' contributions

RSM conducted the experimental work for the study, made significant contributions to the design of the study, and was involved in drafting the manuscript. ATO contributed to the experimental work of the study and made contributions to the manuscript. MSA and PLM both made significant contributions to the study design and were involved in drafting the manuscript. PLM and MSA gave final approval for the manuscript. All authors read and approved the final manuscript.

## References

[B1] Flannery CR Little CB Caterson B Hughes CE Effects of culture conditions and exposure to catabolic stimulators (IL-1 and retinoic acid) on the expression of matrix metalloproteinases (MMPs) and disintegrin metalloproteinases (ADAMs) by articular cartilage chondrocytes Matrix Biol 1999 18 3 225 237 10.1016/S0945-053X(99)00024-4 10429942

[B2] Darling EM Hu JC Athanasiou KA Zonal and topographical differences in articular cartilage gene expression J Orthop Res 2004 22 6 1182 1187 10.1016/j.orthres.2004.03.001 15475195

[B3] Swingler TE Waters JG Davidson RK Pennington CJ Puente XS Darrah C Cooper A Donell ST Guile GR Wang W Degradome expression profiling in human articular cartilage Arthritis Res Ther 2009 11 3 R96 10.1186/ar2741 19549314 PMC2714152

[B4] Pombo-Suarez M Calaza M Gomez-Reino JJ Gonzalez A Reference genes for normalization of gene expression studies in human osteoarthritic articular cartilage BMC Mol Biol 2008 9 17 10.1186/1471-2199-9-17 18226276 PMC2248200

[B5] Pombo-Suarez M Calaza M Gomez-Reino JJ Gonzalez A Practical normalization of mRNA expression in quantitative PCR for cartilage research Osteoarthr Cartil 2009 17 6 818 819 10.1016/j.joca.2008.10.012 19056303

[B6] Ayers D Clements DN Salway F Day PJ Expression stability of commonly used reference genes in canine articular connective tissues BMC Vet Res 2007 3 7 10.1186/1746-6148-3-7 17484782 PMC1884148

[B7] Vandesompele J De Preter K Pattyn F Poppe B Van Roy N De Paepe A Speleman F Accurate normalization of real-time quantitative RT-PCR data by geometric averaging of multiple internal control genes Genome Biol 2002 3 7 RESEARCH0034 12184808 10.1186/gb-2002-3-7-research0034 PMC126239

[B8] Vandesompele geNorm User Manual 2008

[B9] Pfaffl MW Tichopad A Prgomet C Neuvians TP Determination of stable housekeeping genes, differentially regulated target genes and sample integrity: BestKeeper–Excel-based tool using pair-wise correlations Biotechnol Lett 2004 26 6 509 515 15127793 10.1023/b:bile.0000019559.84305.47

[B10] Ohl F Jung M Radonic A Sachs M Loening SA Jung K Identification and validation of suitable endogenous reference genes for gene expression studies of human bladder cancer J Urol 2006 175 5 1915 1920 10.1016/S0022-5347(05)00919-5 16600798

[B11] Brattelid T Winer LH Levy FO Liestol K Sejersted OM Andersson KB Reference gene alternatives to Gapdh in rodent and human heart failure gene expression studies BMC Mol Biol 2010 11 22 10.1186/1471-2199-11-22 20331858 PMC2907514

[B12] Nygard AB Jorgensen CB Cirera S Fredholm M Selection of reference genes for gene expression studies in pig tissues using SYBR green qPCR BMC Mol Biol 2007 8 67 10.1186/1471-2199-8-67 17697375 PMC2000887

[B13] Fundel K Haag J Gebhard PM Zimmer R Aigner T Normalization strategies for mRNA expression data in cartilage research Osteoarthr Cartil 2008 16 8 947 955 10.1016/j.joca.2007.12.007 18258458

[B14] Collins-Racie LA Yang Z Arai M Li N Majumdar MK Nagpal S Mounts WM Dorner AJ Morris E LaVallie ER Global analysis of nuclear receptor expression and dysregulation in human osteoarthritic articular cartilage: reduced LXR signaling contributes to catabolic metabolism typical of osteoarthritis Osteoarthr Cartil 2009 17 7 832 842 10.1016/j.joca.2008.12.011 19217805

[B15] Watson S Mercier S Bye C Wilkinson J Cunningham AL Harman AN Determination of suitable housekeeping genes for normalisation of quantitative real time PCR analysis of cells infected with human immunodeficiency virus and herpes viruses Virol J 2007 4 130 10.1186/1743-422X-4-130 18053162 PMC2216015

[B16] Nishimura M Nikawa T Kawano Y Nakayama M Ikeda M Effects of dimethyl sulfoxide and dexamethasone on mRNA expression of housekeeping genes in cultures of C2C12 myotubes Biochem Biophys Res Commun 2008 367 3 603 608 10.1016/j.bbrc.2008.01.006 18191039

[B17] Ashwell MS O'Nan AT Gonda MG Mente PL Gene expression profiling of chondrocytes from a porcine impact injury model Osteoarthr Cartil 2008 16 8 936 946 10.1016/j.joca.2007.12.012 18276170

[B18] Amin AR Attur M Patel RN Thakker GD Marshall PJ Rediske J Stuchin SA Patel IR Abramson SB Superinduction of cyclooxygenase-2 activity in human osteoarthritis-affected cartilage. Influence of nitric oxide J Clin Invest 1997 99 6 1231 1237 10.1172/JCI119280 9077531 PMC507937

[B19] Geyer M Grassel S Straub RH Schett G Dinser R Grifka J Gay S Neumann E Muller-Ladner U Differential transcriptome analysis of intraarticular lesional vs intact cartilage reveals new candidate genes in osteoarthritis pathophysiology Osteoarthr Cartil 2009 17 3 328 335 10.1016/j.joca.2008.07.010 18775662

[B20] Osterburg HH Allen JK Finch CE The use of ammonium acetate in the precipitation of ribonucleic acid Biochem J 1975 147 2 367 368 1180897 10.1042/bj1470367 PMC1165452

[B21] Erkens T Van Poucke M Vandesompele J Goossens K Van Zeveren A Peelman LJ Development of a new set of reference genes for normalization of real-time RT-PCR data of porcine backfat and longissimus dorsi muscle, and evaluation with PPARGC1A BMC Biotechnol 2006 6 41 10.1186/1472-6750-6-41 17026777 PMC1609116

[B22] Svobodova K Bilek K Knoll A Verification of reference genes for relative quantification of gene expression by real-time reverse transcription PCR in the pig J Appl Genet 2008 49 3 263 265 10.1007/BF03195623 18670063

[B23] Kuijk EW du Puy L van Tol HT Haagsman HP Colenbrander B Roelen BA Validation of reference genes for quantitative RT-PCR studies in porcine oocytes and preimplantation embryos BMC Dev Biol 2007 7 58 10.1186/1471-213X-7-58 17540017 PMC1896162

[B24] Piorkowska K Oczkowicz M Rozycki M Ropka-Molik K Piestrzynska-Kajtoch A Novel porcine housekeeping genes for real-time RT-PCR experiments normalization in adipose tissue: assessment of leptin mRNA quantity in different pig breeds Meat Sci 2011 87 3 191 195 10.1016/j.meatsci.2010.10.008 21041039

[B25] Fitzgerald JB Jin M Dean D Wood DJ Zheng MH Grodzinsky AJ Mechanical compression of cartilage explants induces multiple time-dependent gene expression patterns and involves intracellular calcium and cyclic AMP J Biol Chem 2004 279 19 19502 19511 10.1074/jbc.M400437200 14960571

[B26] Young AA Smith MM Smith SM Cake MA Ghosh P Read RA Melrose J Sonnabend DH Roughley PJ Little CB Regional assessment of articular cartilage gene expression and small proteoglycan metabolism in an animal model of osteoarthritis Arthritis Res Ther 2005 7 4 R852 R861 10.1186/ar1756 15987487 PMC1175037

